# Bond Behavior of Concrete-Filled Steel Tube Mega Columns with Different Connectors

**DOI:** 10.3390/ma15082791

**Published:** 2022-04-11

**Authors:** Robel Wondimu Alemayehu, Jaehoon Bae, Young K. Ju, Min Jae Park

**Affiliations:** 1 School of Civil, Environmental and Architectural Engineering, Korea University, 145 Anam-ro, Seongbuk-gu, Seoul 02841, Korea; robel@korea.ac.kr; 2 Department of Architectural Design, College of Engineering Science, Chonnam National University, Jeonnam 59626, Korea; skycity-bjh@jnu.ac.kr

**Keywords:** mega column, concrete-filled steel tube (CFST), shear studs, rib plate, push out, connectors, finite-element method

## Abstract

Concrete-filled steel tubes (CFSTs) are widely used in construction. To achieve composite action and take full advantage of the two materials, strain continuity at the steel–concrete interface is essential. When the concrete core and steel tube are not loaded simultaneously in regions such as beam or brace connections to the steel tubes of a CFST column, the steel–concrete bond plays a crucial role in load transfer. This study uses a validated finite-element model to investigate the bond-slip behavior between the steel tube and concrete in square CFST mega columns through a push-out analysis of eleven 1.2- × 1.2-m mega columns. The bond-slip behavior of CFST mega columns with and without mechanical connectors, including shear studs, rib plates, and connecting plates, is studied. The finite-element results indicate that the mechanical connectors substantially increased the maximum bond stress. Among the analyzed CFST mega columns, those with closely spaced shear studs and rib plate connectors with circular holes exhibited the highest bond stress, followed by plate connectors and widely spaced shear stud connectors. In the case of shear stud connectors, the stud diameter and spacing influenced the bond behavior more than the stud length. As the stud spacing decreased, the failure mode shifted from studs shearing off to outward buckling of the steel tube.

## 1. Introduction

Concrete-filled steel tubes (CFSTs) are widely used as columns. Construction with CFST columns begins with the erection of hollow-steel-tube columns and framing beams and braces, followed by concrete filling as construction progresses. Thus, the need for formwork is eliminated. In addition, CFST columns offer high strength, fire resistance, ductility, and high energy-dissipation capacity [1]. A steel tube enhances the strength and ductility of infilled concrete by reinforcing and confining it. Simultaneously, concrete prevents buckling of the steel tube and increases the overall stability [1,2]. Test results have shown that the strength and ductility of CFST columns are superior to those of individual components, and the ultimate strength is even greater than the sum of the ultimate strengths of the individual materials [1].

Stress transfer and strain continuity between steel and concrete are required to achieve structural benefits and attain composite action [2,3]. Strain continuity and composite action can be guaranteed when the concrete and steel tube are simultaneously loaded [4]. However, when structural members, such as beams and braces, are attached to a steel tube, sufficient steel–concrete bond stress is required to ensure force transfer and strain continuity [2].

The bond behavior of CFST columns has been widely studied [5,6,7,8,9,10]. The push-out test introduced by Dowling [11] is mainly used to study the bond behavior of CSFT columns. Numerous push-out tests conducted indicate that the bond stress behavior depends on the interface type, concrete grade, shape, and size of the cross-section, and the source of the steel–concrete bond is primarily a result of the chemical adhesion of the cement gel, mechanical connectors at the interface, and frictional force [12,13].

**Figure 1:**
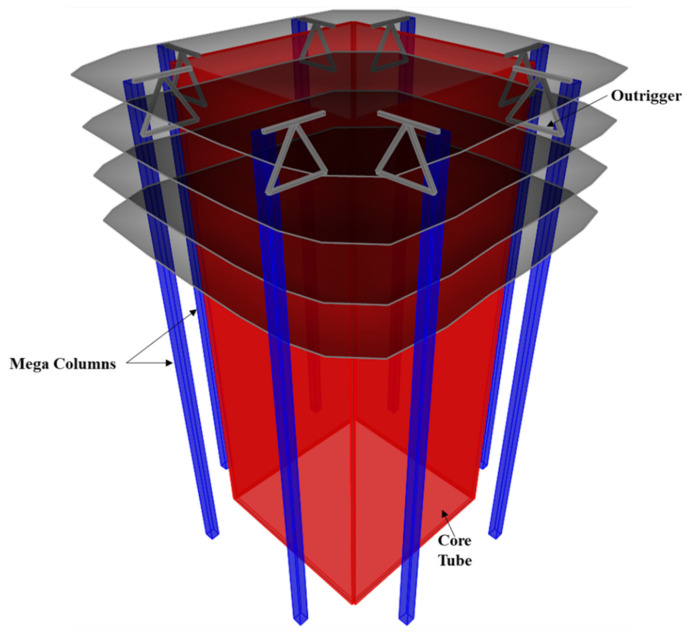
Mega columns in typical high-rise construction.

Although numerous CFST bond behavior studies have been conducted, most previous push-out tests have been conducted on small cross-sections [13] and may not represent CFST mega columns typically used in high-rise construction. Figure 1 shows a typical super-structure system incorporating mega columns, and the cross-section of mega columns typically exceeds 1 m. In this study, the bond stress behavior of 1.2- × 1.2-m mega CFST square columns was explored through push-out analysis using a validated finite-element model, and comparison with current practice codes was made.

## 2. Parametric Finite-Element Analysis

The parametric variables cover a comprehensive set of connector types: shear stud connectors, rib plate connectors, rib plate connectors with circular holes, rib plate connectors combined with shear studs, and rib plate connectors in combination with connecting plates. To obtain a realistic and representative mega CFST column size and the material grades, the adapted mega CFST column was extracted from a skyscraper project. The steel tube in the adopted CFST mega column was 1200 × 1200 mm and had a wall thickness of 25 mm. Moreover, the steel tube and connecting ribs were fabricated using SM460 steel with a nominal yield and ultimate strength of 460 and 570 MPa, respectively. The steel grade used for all other steel components was SM355 with a specified minimum yield and ultimate strength of 355 and 470 MPa, respectively. The shear studs used as connectors were HS1 studs with a yield and ultimate strength of 235 and 400 MPa, respectively.

**Figure 2:**
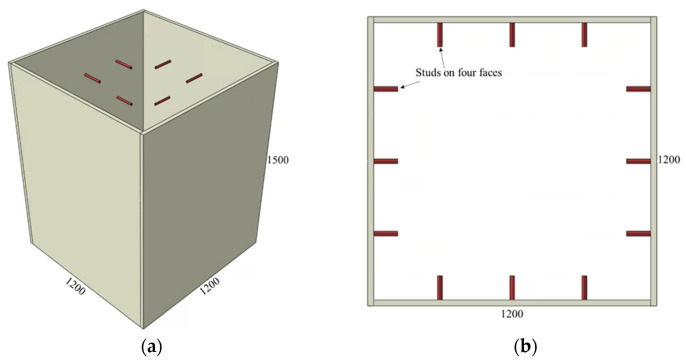
CFST column with shear studs on all faces: (**a**) 3D view and (**b**) top view.

**Figure 3:**
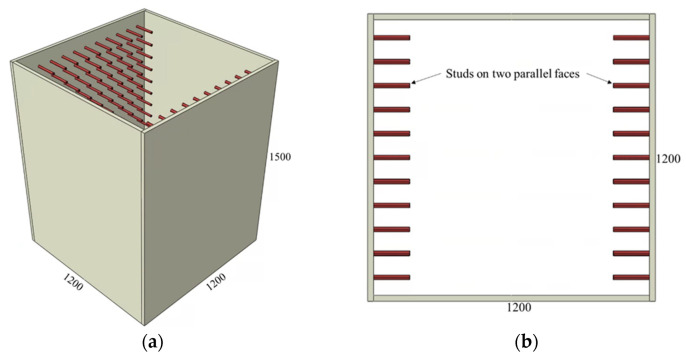
CFST column with shear studs on two parallel faces: (**a**) 3D view and (**b**) top view.

**Figure 4:**
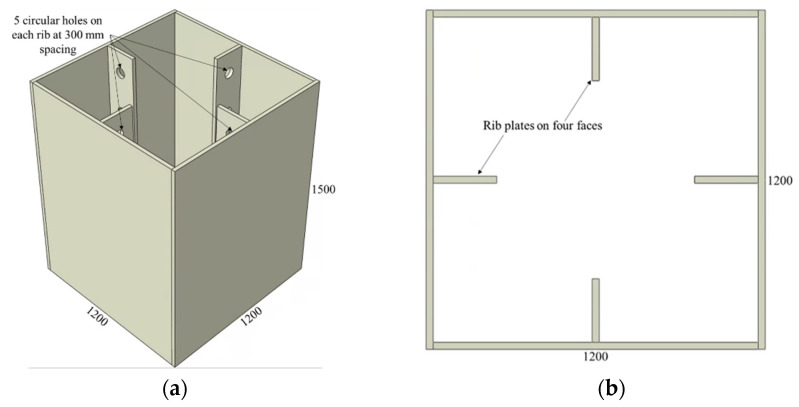
CFST column with rib plate connectors: (**a**) 3D view and (**b**) top view.

**Figure 5:**
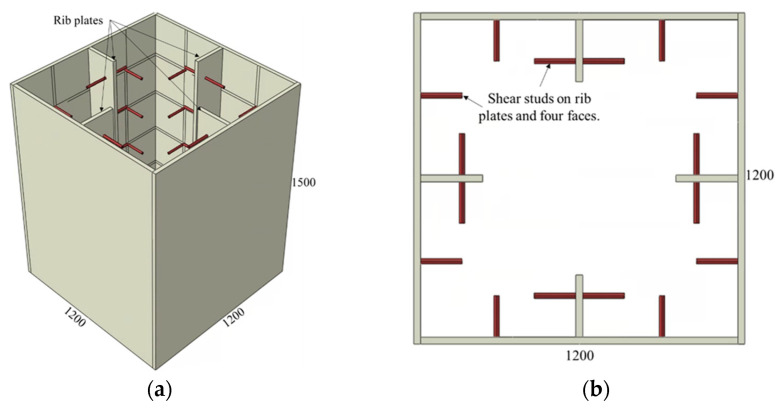
CFST column with rib plate and shear stud connectors: (**a**) 3D view and (**b**) top view.

**Figure 6:**
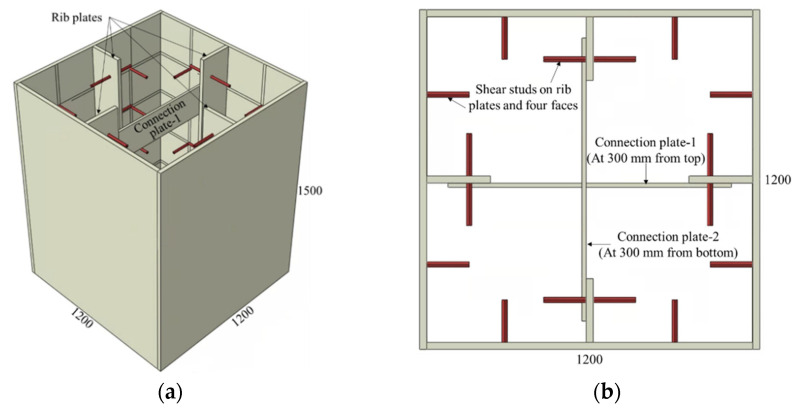
CFST column with rib plate, shear stud, and connecting plates: (**a**) 3D view and (**b**) top view.

The adopted connector types and variations of the investigated parameters are summarized in Figure 2, Figure 3, Figure 4, Figure 5 and Figure 6 and Table 1, Table 2 and Table 3. The adopted connector types are grouped into five, and the first group includes shear stud connectors. Figure 2 and Figure 3 depict a CFST mega column with shear stud connectors on four and two parallel faces, respectively. The varied parameters for the stud connectors include the stud spacing, stud diameter, and stud length. The adopted variations are summarized in Table 1, with the model names indicating the stud arrangement and the values of the investigated parameters. S4 and S2 at the beginning of the model names indicate the shear studs on all four faces and two parallel faces, respectively. The numbers following Sp, D, and L in the model names represent stud spacing, stud diameter, and stud length in millimeters, respectively. For example, S4-Sp300D19L100 means studs on all four faces at a spacing of 300 mm, and each stud has a diameter of 19 mm and length of 100 mm.

Similarly, the CFST columns with rib plate connectors are shown in Figure 4, and the values of the investigated parameters are listed in Table 2. The names of CFST columns with rib plate connectors start with an R, followed by the designation of the hole diameter provided on the ribs, as illustrated in Figure 3. The circular holes in all rib plates are spaced at 300 mm, with the first hole starting 150 mm from the top. A rib plate connector with no holes is also included in this category and designated as having a zero-hole diameter (R-HD0).

The final group of CFST mega columns analyzed incorporates a combination of stud connectors, rib plates, and connection plates, as illustrated in Figure 5 and Figure 6. The geometric details of these models are summarized in Table 3, with the start of the model names indicating the combination of connectors used. The model S4-R represents a CFST mega column with shear stud connectors on four faces together with rib connectors. Likewise, the model S4-R-Cp indicates that studs on four faces, rib plates, and connection plates were used in combination as a connector. The stud spacing, diameter, and length in these two models were 300, 19, and 150 mm, respectively.

### 2.1. Finite-Element Modeling

**Figure 7:**
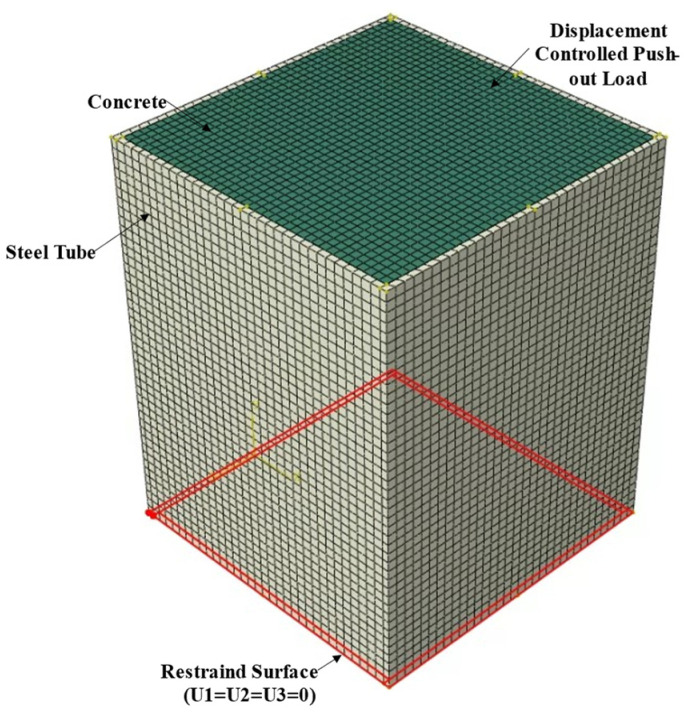
Finite-element mesh and boundary conditions.

Finite-element models of the mega CFST columns were formulated using ABAQUS version 2017 [14]. The geometric and material nonlinearities were considered in this analysis. Push-out analysis was conducted by restraining the three translational degrees of freedom of the steel tube base and applying a displacement control load at the top surface of the concrete, as shown in Figure 7. Since only the steel tube at the bottom is supported, and the load is applied to the top surface of the concrete, the applied load is transferred from the concrete to the steel tube via bond stress at the steel–concrete interface.

The concrete and studs were meshed using reduced integration eight-node linear brick elements with reduced integration and hourglass control, i.e., C3D8R. The steel components were meshed using reduced integration 20-node quadratic brick elements to capture geometric nonlinearities better. Hexagonal elements with an average size of 30 mm were used to mesh the finite-element models. The finite-element model mesh is shown in Figure 7.

The material property of the steel components was modeled as bilinear kinematic hardening using the respective yield and ultimate strength of the steel components. The ultimate strain was assumed to be 0.2. For the elastic range, an elastic modulus and Poisson’s ratio of 200 GPa and 0.3, respectively, were adopted.

**Figure 8:**
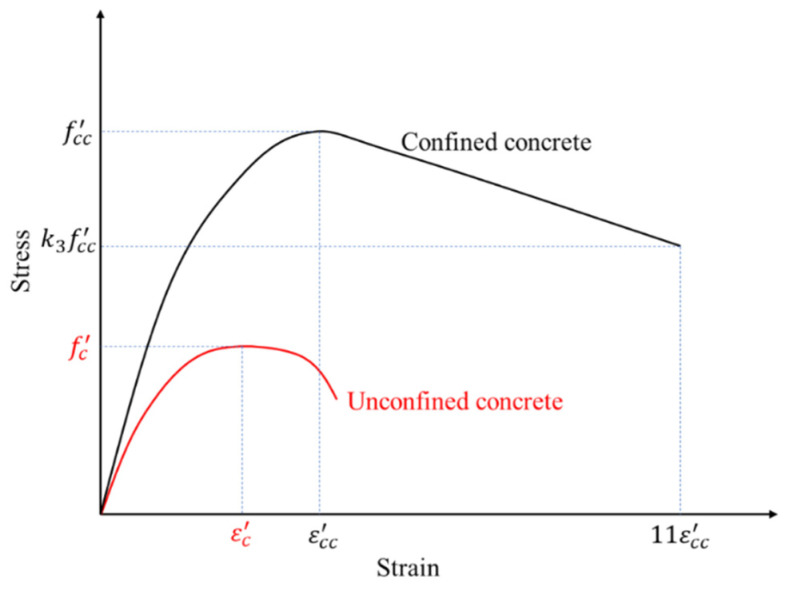
Uniaxial stress–strain curve for concrete.

The uniaxial compressive stress–strain relationship of the confined C70 concrete was modeled using the Drucker–Prager hardening rule by utilizing the material constitutive model for confined concrete [15]. The adopted constitutive model is illustrated in Figure 8. The unconfined concrete stress–strain relation along with the compressive strength $f_{c}^{\prime }$ and the corresponding strain $\varepsilon_{c}^{\prime }$ are shown in red. Here, $\varepsilon_{c}^{\prime }$ was taken as 0.003 in the analysis based on the ACI 318 [16] recommendation. When concrete is subjected to confining pressure, the compressive strength $f_{cc}^{\prime }$ and the corresponding strain $\varepsilon_{cc}^{\prime }$ are higher than those of unconfined concrete, as illustrated in Figure 8 [15]. The confined strength and the corresponding strain are related by Equations (1) and (2), respectively [17].

(1) $$f_{cc}^{\prime }=f_{c}^{\prime }+k_{1}f_{1}$$

(2) $$\varepsilon_{cc}^{\prime }=\varepsilon_{c}^{\prime }\left(1+k_{2}\frac{f_{1}}{f_{c}^{\prime }}\right)$$

where $k_{1}$ and $k_{2}$ are constants determined experimentally [15]. The constants $k_{1}$ and $k_{2}$ were set as 4.1 and 20.5, respectively, based on the study by Richart et al. [18]. Here, $f_{1}$ denotes the confining pressure, which was taken as zero for the size and shape of the mega column analyzed based on the empirical formulation from a previous study [15]. In other words, the compressive strength and corresponding strain did not increase owing to confinement. Only the strength degradation, as illustrated in Figure 8, was altered due to confinement. The material degradation parameter $k_{3}$ depends on the shape and width-to-thickness ratio of the confining steel tube, and it was taken as 0.49 based on the empirical formulation by Hu et al. [15].

(3) $$f_{c}=\frac{E_{c}\varepsilon_{c}}{1+\left(R+R_{E}-2\right)\left(\frac{\varepsilon_{c}}{\varepsilon_{cc}^{\prime }}\right)-\left(2R-1\right){\left(\frac{\varepsilon_{c}}{\varepsilon_{cc}^{\prime }}\right)}^{2}+R{\left(\frac{\varepsilon_{c}}{\varepsilon_{cc}^{\prime }}\right)}^{3}}$$

(4) $$R=\frac{R_{E}\left(R_{\sigma }-1\right)}{{\left(R_{\varepsilon }-1\right)}^{2}}-\frac{1}{R_{\varepsilon }},\;R_{E}=\frac{E_{c}\varepsilon_{cc}^{\prime }}{f_{cc}^{\prime }}$$

(5) $$E_{c}=4700\sqrt{f_{cc}^{\prime }}$$

When plastic deformation occurs, certain parameters should dictate the yield surface’s expansion. Therefore, once the confined compressive strength ($f_{cc}^{\prime })$ and corresponding strain ($\varepsilon_{cc}^{\prime }$) were determined, the uniaxial compressive stress–strain relationship was formulated using Equations (3)–(5) [15], where $f_{c}$, $\varepsilon_{c}$, and $E_{c}$ represent the uniaxial compressive stress, strain, and elastic modulus, respectively. $R_{E}$ in Equation (4) represent the ratio of the initial modulus to the secant modulus at $f_{cc}^{\prime }$. Equation (5), adapted from ACI 318 [16], was used to calculate the initial elastic modulus. The constants $R_{\sigma }$ and $R_{\varepsilon }$ are parameters dependent on the descending branch of the stress–strain curve and highly test dependent. In this study, $R_{\sigma }$ and $R_{\varepsilon }$ were taken as 4 based on Hu et al. [19].

The steel–concrete contact, i.e., the contact between the concrete and the inside walls of the steel tubes and ribs, was modeled as a hard contact with no penetration in the normal direction. The contact behavior in the tangential direction was modeled with surface-based cohesive elements defined by a traction separation relation on the tube-concrete interface with a damage mechanism [14,20]. In this study, the stiffness of the cohesive elements in the two tangential directions was assumed to be uncoupled and equal. Moreover, a cohesive element stiffens of 0.55 MPa/mm was determined to reflect the initial stiffness of the push-out test result by Tao et al. [13]. The damage initiation criterion was defined by limiting the maximum slip before decohesion commences, and when the damage initiation criterion is met, the cohesive element is degraded. The maximum slip before decohesion was determined to be 0.072 mm based on the test results of Tao et al. [13]. Following the decohesion, the tangential contact property was defined using a penalty friction formulation with a coefficient of friction of 0.25.

The interaction of the concrete with the stud connectors and connecting plates was modeled using the ABAQUS embedded region constraint [14], with the concrete as a host region and the studs and connecting plates as an embedded region to make force transfer possible.

CFST column push-out tests conducted by Tao et al. [13] showed that CFST columns with shear studs failed because the studs sheared off at the stud–tube weld while the weld remained intact. To incorporate this phenomenon in the finite-element model, the stud–steel-tube weld was modeled as a cohesive element with a 650 MPa/mm stiffness, and decohesion initiates after a 1.35-mm slip. The cohesive element stiffness and decohesion slip were calibrated to match the experimental observations by Tao et al. [13].

### 2.2. Validation of Finite-Element Model

**Figure 9:**
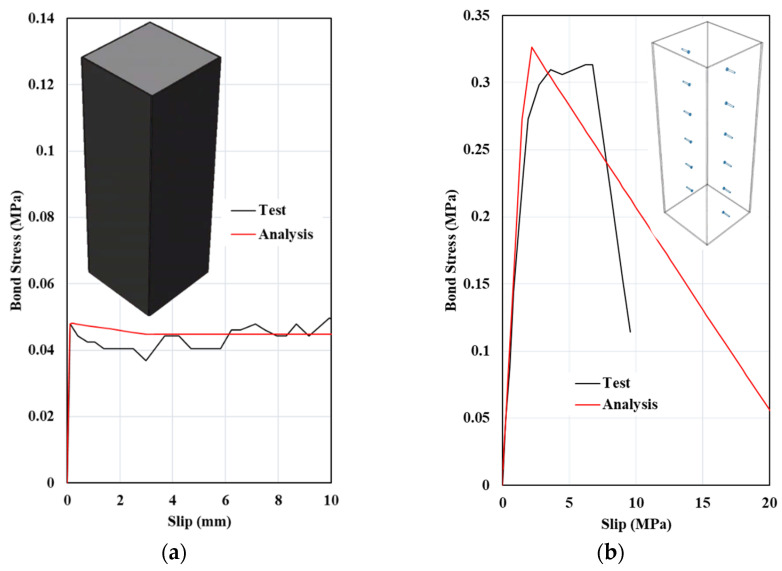
Comparison of test and finite-element method results: (**a**) square CFST without stud connectors and (**b**) square CFST with stud connectors.

The accuracy of the finite-element modeling assumptions for the CFST mega column was validated using the experimental results obtained by Tao et al. [13]. In Figure 9, the finite-element prediction and test results of a CFST column without a connector (nominal interface) and with shear stud connectors are compared. As shown in Figure 9a, the adopted cohesive element formulation reflects the bond stress–slip relation when mechanical connectors are not used. Moreover, as shown in Figure 9b, the finite-element model reflects the stud shearing-off phenomenon observed in the tests, and the bond stress–slip relation agrees well with the test result until the maximum bond stress develops.

## 3. Discussion of Results

**Figure 10:**
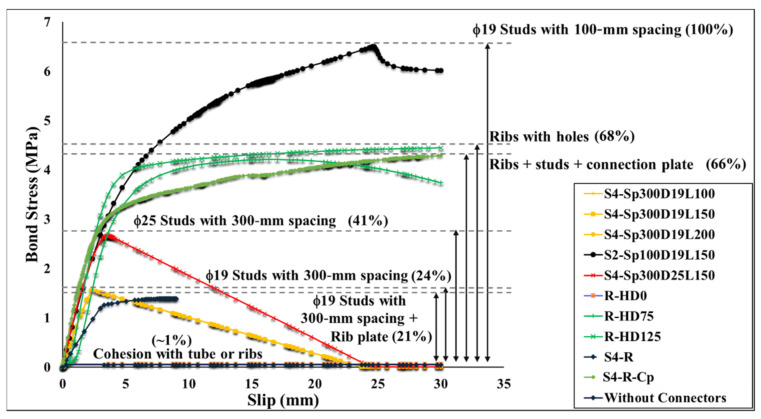
Bond stress–slip curve.

**Figure 11:**
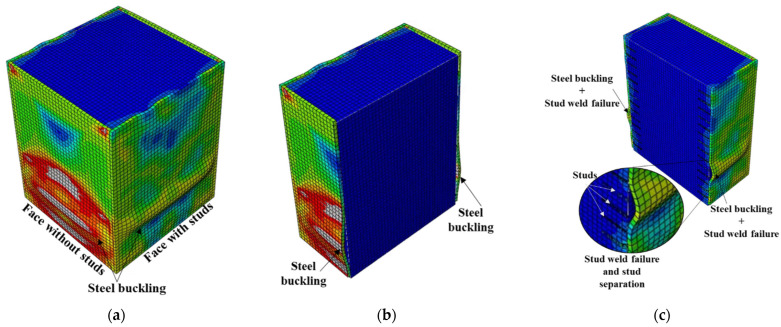
Failure mode of S2-Sp100D19L150 (closely spaced stud on two parallel faces): (**a**) steel tube buckling, (**b**) outward buckling of faces without studs, and (**c**) outward buckling and stud weld failure.

**Figure 12:**
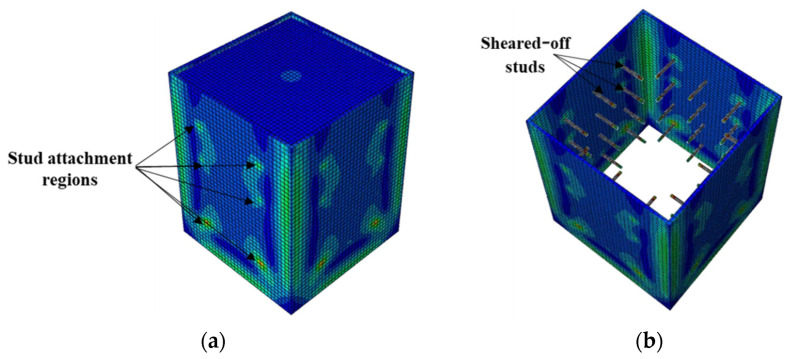
Typical failure mode of CFST columns with shear studs on four faces: (**a**) stress distribution and (**b**) failure mode.

**Figure 13:**
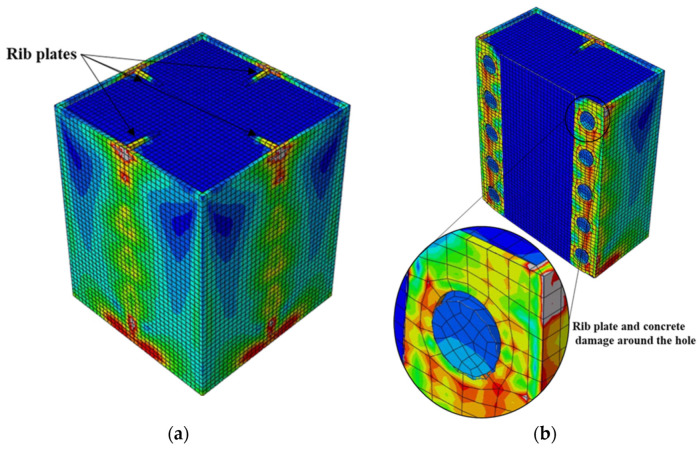
Typical failure mode of CFST columns having rib plates with circular hole connectors: (**a**) stress distribution and (**b**) failure mode.

**Figure 14:**
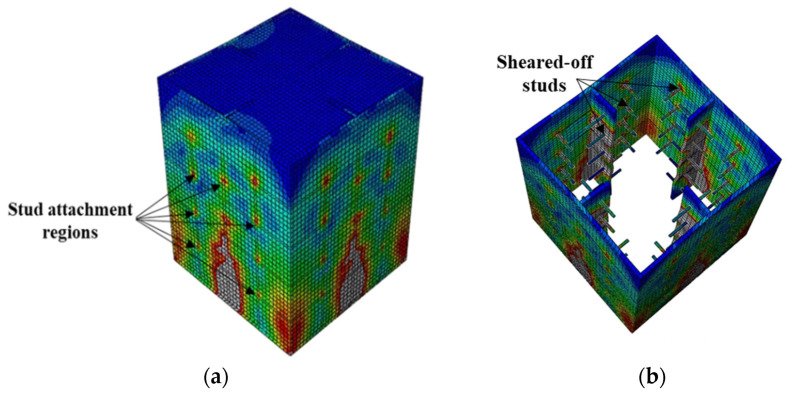
Typical failure mode of CFST columns with shear studs and rib connectors: (**a**) stress distribution and (**b**) failure mode.

**Figure 15:**
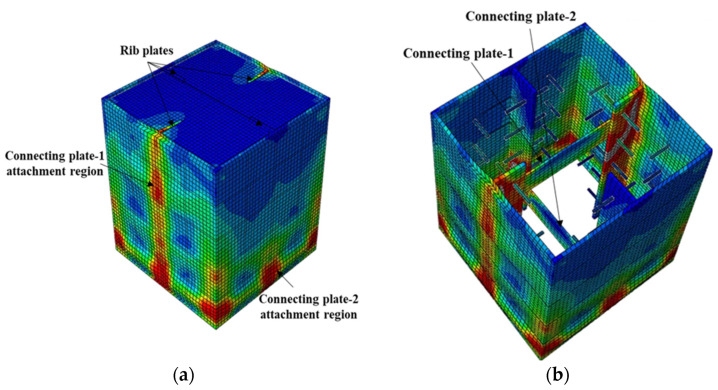
Failure mode of S4-R-Cp (studs, rib plates, and connecting plates): (**a**) stress distribution and (**b**) stress distribution on inner face.

**Figure 16:**
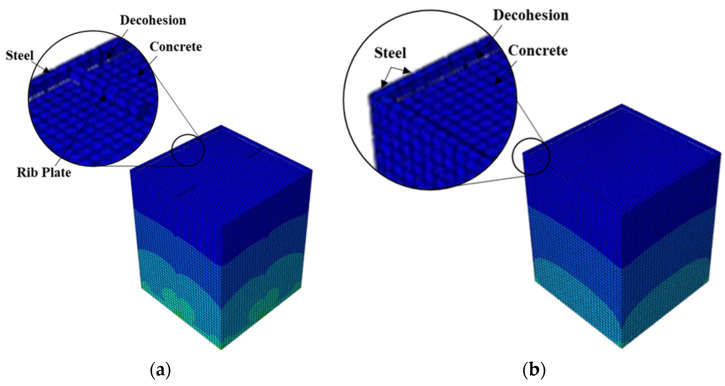
Typical failure mode of CFST columns relying on cohesion only: (**a**) R-HD0 and (**b**) without connectors.

The bond stress–slip relations obtained from the finite-element analysis and the different observed failure modes are illustrated in Figure 10, Figure 11, Figure 12, Figure 13, Figure 14, Figure 15 and Figure 16. Here, the bond stress is calculated as the ratio of the applied load to the steel tube—concrete contact area, and the slip is calculated as the vertical displacement of the concrete relative to the steel tube. As the graph in Figure 10 reveals, the S2-Sp100D19L150 specimen developed the highest bond stress compared with the remaining CFST columns analyzed. The S2-Sp100D19L150 model failed because of outward buckling of the steel tube accompanied by shear stud failure around the buckled region, as shown in Figure 11. Compared with the steel tube face with studs, the stress and buckling deformation were more pronounced on the steel tube face without stud connectors.

In contrast, the stress distribution was uniform in the S4 models with studs on all the faces of the steel tube. All the S4 models analyzed failed by shear-stud–steel-plate connection failure, consistent with the experimental observations of Hu et al. [15]. In the S4 models with the same stud spacing and stud diameter, varying the stud length from 100 to 200 mm did not affect the bond stress–slip behavior. In contrast, increasing the diameter of the studs by 31.5% while keeping the spacing and length constant resulted in a 70.5% increase in the maximum bond strength. As shown in Figure 10, the maximum bond stresses achieved by the S4 models with diameters of 19 and 25 studs were 41% and 24%, respectively, compared with the S2 model. The S2 model showed higher maximum bond stress than the S4 models because of the many studs. Forty-eight studs were used in each of the S4 models, whereas 308 studs were used in the S2 model.

The S2 specimen failed by steel tube outward buckling and showed considerable strength and stiffness beyond the proportionality limit. However, the bond stress was lost in the S4 models with fewer studs once the maximum bond stress was reached. These results are consistent with the experimental observations [15].

Next to the S2 model, the rib plate connectors with circular holes (R models) exhibited the highest bond stress. The two R models with circular holes displayed distinct bond stress–slip relationships depending on the hole diameter. Nonetheless, both achieved a maximum bond stress of 68% that of S2, as illustrated in Figure 10. The bond stress in the R-HD75 model started degrading after attaining the maximum bond stress because of the plastic deformation of the rib plate and concrete damage around the circular holes, as shown in Figure 13. Conversely, such bond stress degradation was not observed in the R-HD125 model up to a 30-mm slip, and a 30-mm slip was the maximum considered in the analysis.

Incorporating shear studs together with rib plates, as in S4-R, gave a maximum bond stress comparable to the S4 models by achieving a maximum bond stress 21% that of the S2 model, as shown in Figure 10. Despite the maximum bond stress being comparable, the bond stress–slip relationship and the stress level in the steel tube differ. The initial stiffness of the bond stress–slip curve was lower than that of the S4 models. Moreover, the steel tube stress in the S4-R was higher than that in the S4 models. Similar to the S4 models, the S4-R model failed because of shear studs shearing off the steel tube and rib plates.

Connecting plates with rib plates and shear studs, as in S4-R-Cp, improved the bond stress performance beyond the proportionality limit with no bond stress degradation up to a 30-mm slip. The maximum bond stress achieved was 66% of that of the S2 model. As shown in Figure 10, the maximum bond stress reached by the S4-R-Cp model is comparable to that of the R models with circular holes. However, S4-R-Cp reached the proportionality limit at bond stress which was 45.3% that of the R-HD125 model.

Finally, the R-HD0 model and the model without connectors exhibited the lowest bond stresses, as illustrated in Figure 10. The bond stress in these two models relied on the cohesion between the concrete and steel tube/rib plates. As a result, the bond stress in these models was lost when decohesion occurred, as shown in Figure 16. The addition of rib plates increased the steel–concrete contact area; however, the bond strength improvement was negligible. Table 4 summarizes the key parameters obtained from the analysis and the failure modes.

The efficiency of each model was examined by comparing the maximum bond stress and volume of connectors used. As shown in Table 4, the S4 models with 19 mm stud diameter exhibited similar maximum bond stress despite the stud length variation. On the contrary, increasing the cross-sectional stud area by 1.73 times while keeping the stud length and spacing constant resulted in a maximum bond stress increase by a factor of 1.72. The most efficient connector type with the highest bond stress per volume of connector used was the stud connector distributed on four faces (S4), followed by stud connectors on two parallel faces (S2), rib plate connectors with circular holes, connecting plate, rib plate with studs (S4R), and rib plate without holes, in that order.

The maximum bond strengths obtained from the analysis were compared to the requirements of current practice codes. The minimum bond strength requirement of the Chinese code DBJ/T 13-54-2010 [21] and Japanese code AIJ [22] is 0.225 MPa. The British code BS 5400-5 [23] and European code EN1994 [24] require a minimum bond strength of 0.4 and 0.55 MPa, respectively. Thus, all the analyzed mega columns with mechanical connectors satisfied the requirements of the four codes [21,22,23,24], whereas the two models that relied on cohesion failed to fulfill the requirements.

## 4. Conclusions

The bond strength between the steel tubes and concrete in concrete-filled steel tube mega columns was investigated using a validated finite-element model. The finite-element investigation included 11 identical 1.2- × 1.2-m CFST mega columns with different types and arrangements of steel tubes and concrete connectors. The following conclusions can be drawn based on the finite-element results.

1. The maximum bond stress achieved by the CFST mega column models with mechanical connectors satisfies the minimum requirements of DBJ/T, AIJ, BS5400-5, and EN 1994 while the minimum requirement of the four codes was not met by the mega columns that relied on cohesion only.
2. Closely spaced stud connectors (S2-Sp100D19L150) exhibited the highest bond stress, followed by rib connectors with circular holes (R-HD125) and connection plates that run across the CFST (S4-R-Cp).
3. Although S2-Sp100D19L150 exhibited high bond stress, nonlinearity started early compared with other models that exhibited high bond stress.
4. The rib plate connectors with circular holes exhibited both a high maximum bond stress and a high bond stress before losing linearity.
5. Increasing the stud length had a negligible effect on the bond performance for the same number of studs. However, increasing the stud diameter resulted in improved bond performance.
6. The use of closely spaced studs, rib plates with circular holes, and connecting plates that run between the parallel walls of the CFST resulted in a considerable slip before the strength degradation commenced. Moreover, the bond stiffnesses of these three connector types were on the same order.
7. Increasing the circular hole diameter from 75 to 125 mm in the CFST columns with rib connectors improved the bond strength, stiffness, and maximum bond stress before the proportionality limit.
8. The CFST columns that relied solely on the cohesion between steel and concrete (CFST without connectors and R-HD0) showed the poorest performance.
9. The stud connectors followed by the rib plate connectors with circular holes were the most efficient with respect to maximum bond stress per connector unit volume.

## Figures and Tables

**Table 1:** List of finite-element models with shear stud connectors.

Model Name	Stud Spacing	Stud Diameter	Stud Length	Remarks
S4-Sp300D19L100	300	19	100	Studs on four faces of the CFST
S4-Sp300D19L150		19	150	
S4-Sp300D19L200		19	200	
S4-Sp300D25L150		25	150	
S2-Sp100D19L150	100	19	150	Studs on two parallel faces of the CFST

All dimensions are in millimeters.

**Table 2:** List of finite-element models with rib plate connectors.

Model Name	Rib Thickness	Rib Depth	Rib Hole Diameter
R-HD0	25	225	-
R-HD75			75
R-HD125			125

All dimensions are in millimeters.

**Table 3:** List of finite-element models with rib plate and shear stud connectors.

Model Name	Rib Thickness	Rib Depth	Stud Spacing	Stud Diameter	Stud Length	Connecting Plate Thickness
S4RSp300D19L150	25	225	300	19	150	-
S4RCpSp300D19L150						15

All dimensions are in millimeters.

**Table 4:** Summary of finite element analysis results.

Model Name	Total Connector Volume (×10^6^ mm^3^)	Max Bond Stress (MPa)	Slip at Max. Bond Stress (mm)	Initial Bond Stress–Slip Slope (MPa/mm)	Bond Stress at Proportionality Limit (MPa)	Failure Mode
S4-Sp300D19L100	1.36	1.56	2.35	0.68	1.56	Stud–plate weld failure
S4-Sp300D19L150	2.04	1.55	2.26	0.69	1.55	
S4-Sp300D19L200	2.72	1.56	2.35	0.68	1.56	
S4-Sp300D25L150	3.53	2.66	3.54	1.11	1.60	
S2-Sp100D19L150	13.10	6.50	24.65	1.02	1.59	Outward buckling of steel tube and stud–plate weld failure
R-HD0	33.75	0.06	3.60	0.16	0.06	Steel–concrete bond failure (decohesion)
R-HD75	31.54	4.21	16.35	1.16	2.51	Damage of concrete and rib plate near hole
R-HD125	27.61	4.45	30.00	1.41	2.82	No strength degradation up to 30-mm slip
S4-R	36.47	1.39	19	0.42	0.98	Stud–plate weld failure
S4-R-Cp	42.77	4.29	30.00	1.27	1.94	No strength degradation up to 30-mm slip
Without connector	-	0.05	3.60	0.16	0.05	Steel–concrete bond failure (decohesion)

## Data Availability

Not applicable.
